# Birth weight and heart rate autonomic recovery following exercise in healthy term-born adults

**DOI:** 10.1038/s41598-020-80109-3

**Published:** 2021-01-13

**Authors:** Giovanna de Paula Vidigal, David M. Garner, Amanda N. Akimoto, Vitor E. Valenti

**Affiliations:** 1 Autonomic Nervous System Center (CESNA), São Paulo State University, UNESP, Av. Hygino Muzzi Filho, 737, Mirante, Marilia, Presidente Prudente, SP 17525-900 Brazil; 2 Cardiorespiratory Research Group, Department of Biological and Medical Sciences, Faculty of Health and Life Sciences, Oxford Brookes University, Headington Campus, Gipsy Lane, Oxford, OX3 0BP UK

**Keywords:** Cardiology, Medical research

## Abstract

The impact of birth weight (BW) on adult health has been studied, related to the autonomic nervous system, and implicated in cardiovascular risk. We investigated cardiorespiratory and heart rate (HR) autonomic recovery after moderate effort in healthy term-born adults with different BWs. We studied 28 healthy physically active women aged between 18 to 30 years split equally into two groups according to BW: G1 (n = 14), BW between 2500 g and 3200 g and G2 (n = 14), BW > 3200 g. The groups remained seated at rest for 15 min, followed by aerobic exercise on a treadmill (five minutes at 50–55% of maximum HR and 25 min 60–65% of maximum HR) and then remained seated for 60 min during recovery from the exercise. Cardiorespiratory parameters and HR variability (HRV) [RMSSD, HF (ms^2^)] were assessed before and during recovery from exercise. In G1, HR was increased from 0 to 20 min after exercise whilst in G2 HR was higher from 0 to 7 min following exercise. In G1, short-term HRV was increased from 5 to 10 min after exercise but in G2 it recovered prior to 5 min following effort. In conclusion, healthy term-born women with low normal BW present slower HR autonomic recovery after exercise.

## Introduction

Cardiovascular diseases are currently recognized as a major cause of death globally. In 2013, almost 32% of deaths worldwide were attributable to cardiovascular diseases^1^. Cardiovascular risk can be evaluated via heart rate (HR) variability (HRV), which assesses instabilities between inter-beat intervals (IBI)^2^. Decreased HRV is correlated to an increased risk of cardiovascular diseases^3^.

According to a systematic review regarding HRV in newborns^4^, it was detected that the autonomic nervous system begins to develop in the third trimester of pregnancy, and continues to develop even after birth^5^. In a preterm child (< 37 full weeks of gestation), the autonomic nervous system maturation is momentarily impaired. A longitudinal comparative study found greater HRV in term infants compared to preterm infants, indicating that prematurity has an impact on the parasympathetic nervous system^6^. Likewise, a recent study revealed reduced HR vagal regulation in healthy children with normal low BW, signifying an increased risk of cardiovascular and metabolic diseases in this population^7^.

Thus, the literature has investigated the impact of birth weight (BW) on adulthood^8–10^. Persons with normal low BW (< 2500 g) have a tendency to present higher risk of cardiovascular diseases^11–13^ and insulin resistance^14^ in adulthood. In contrast, Perkiömäki et al.^15^ compared adults with BW < 2500 g with BW > 4000 g and revealed that middle-aged men with normal high BW may have greater cardiovascular risk than in adults with normal low BW.

Recently, Tian et al.^16^, evaluated the relationship between BW and waist circumference on cardiovascular disease in adults. A cohort of 2200 persons was randomly selected from 18,000 eligible permanent Chinese inhabitants. Their key outcome revealed that normal low BW may a predictor of cardiovascular disease in adults.

In addition, recent studies described the adverse effect of normal low BW (< 2500 g) on autonomic function in adults born preterm^17,18^. The mentioned studies employed autonomic recovery following exercise, and this is a dependable technique when estimating cardiovascular risk. Slower recovery after exercise cessation is related to elevated cardiovascular risks^19^.

Yet, it is unclear if there is any change between healthy term-born adults with different BW values. Bearing this in mind, we propose the following question: does healthy term-born adults with normal high BW (> 3200 g) display improved autonomic function and reduced levels of cardiovascular disease? Therefore, we evaluated cardiorespiratory and HR autonomic recovery following moderate exercise in healthy term-born adults with different BW. We intended to confirm whether healthy adult term-born with normal high BW presented faster recovery and, in a positive case, to recommend that BW > 3200 g is advantageous over BW < 3200 g.

## Method

### STROBE Guidelines

This study followed the STROBE (STrengthening the Reporting of OBservational studies in Epidemiology) guidelines. Our investigation describes information regarding setting, variables, study design, participants, measurements, data sources, quantitative variables description, statistical methods and potential sources of bias.

### Population study and eligibility criteria

**Figure 1:**
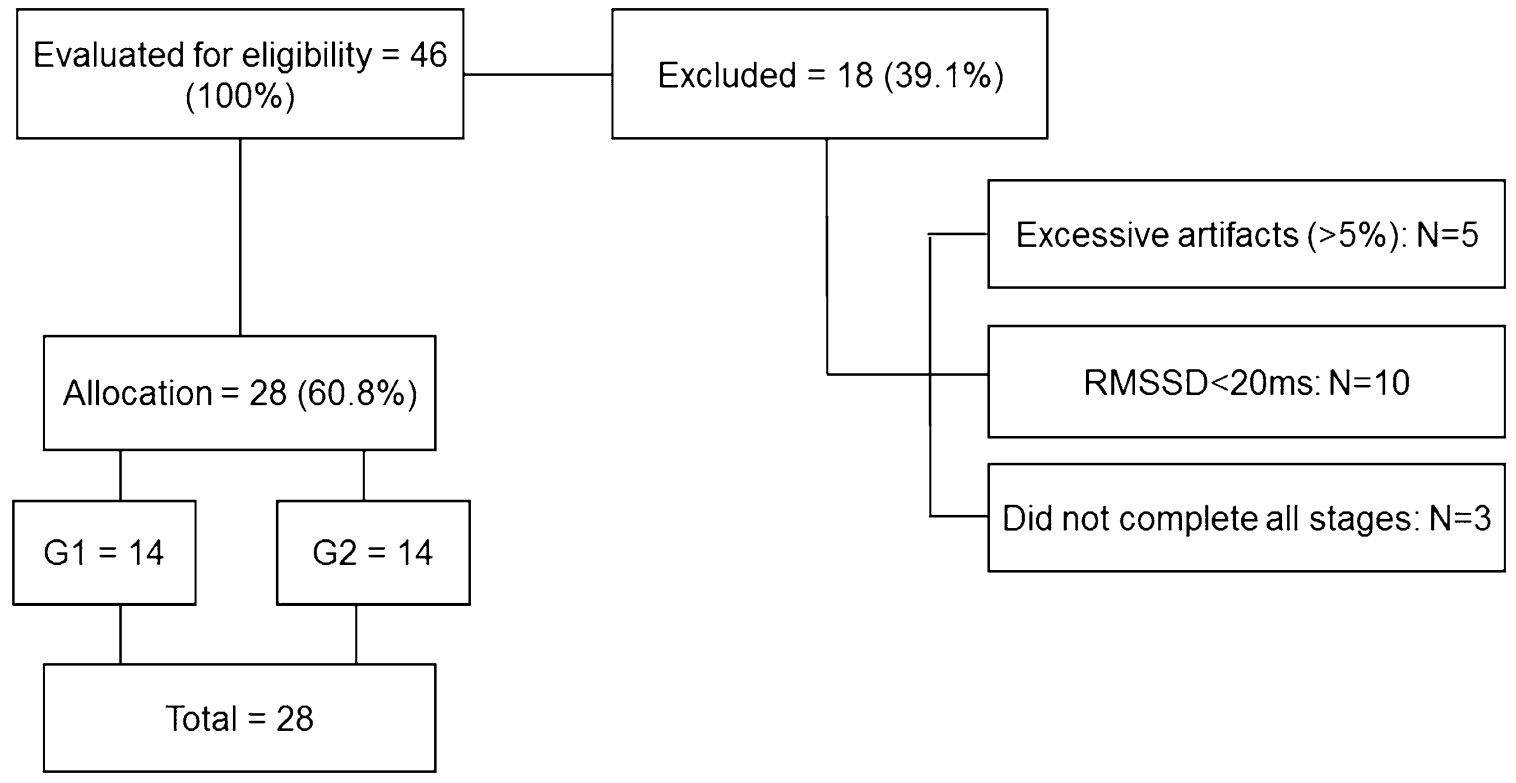
Flowchart.

Initially, we examined 46 healthy term born adult females with regular menstrual cycles enrolled at the Sao Paulo State University, Marilia, Brazil. After excluding subjects as a consequence of high artifacts in HR recording (> 5%), low resting RMSSD (root-mean square of differences between adjacent normal IBI < 20 ms) and not completing all required stages of the experimental protocol; the final sample became 28 subjects (Fig. 1).

We excluded subjects with birth weight < 2000 g, body mass index (BMI) > 25 kg/m^2^ or BMI < 18.5 kg/m^2^, subjects with anxiety, depression, cardiorespiratory, neurological, musculoskeletal, renal, metabolic, endocrine and other known or reported deficiencies that prohibited the performance of the specified protocols. Subjects under pharmacological treatments, smokers, alcoholics, sedentary and inadequately active individuals according to the International Physical Activity Questionnaire (IPAQ)^20^ were also omitted.

Subjects whose mother reported gestational complications were excluded. All were breast-fed for no less than 6 months of extrauterine life and were born via an elective cesarean section.

We excluded women between the 10th and 15th days and between the 20th and 25th days of the menstrual cycle to remove potential influences of their luteal and follicular phase, respectively^21^.

The subjects were split equally into two groups, according to birth weight: (G1) birth weight between 2400 and 3200 g, (n = 14) and; (G2) birth weight between 3200 and 4500 g, (n = 14). We selected these diverse weights so as to investigate if birth weight > 3200 g is healthier than a birth weight < 3200 g.

### Ethical approval and informed consent

All procedures were performed in accordance with the 466/2012 resolution of the National Health Council of December 12th 2012 and all subjects signed a confidential informed consent letter. All experimental protocols were inspected and approved by the Research Ethics Committee in Research of UNESP/Marilia through the Brazilian online platform (Number 5406).

### Study design and setting

This is a prospective, observational and analytical study completed at the Autonomic Nervous System Center, UNESP, Marilia, SP, Brazil.

### Potential sources of bias

With the purpose of governing potential sources of bias, all procedures were performed in a noiseless room with temperature between 21 °C and 25 °C, humidity between 40 and 60% and between 17:00 and 22:00 to standardize and avoid circadian influences^22^. The subjects were instructed to avoid drinking alcohol or caffeinated drinks and to not perform exhaustive exercise 24 h before the protocols. Subjects were dressed in appropriate and comfortable clothing to permit the required physical effort and they were advised to eat a light meal only 2-h before the procedures.

The descriptive profile of the individuals was demarcated to describe the sample, reduce the unpredictability of the variables, enhance reproducibility, improve physiological and clinical interpretation. We measured age, systolic (SBP) and diastolic blood pressures (DBP), mass (kg), height (m), fat percentage and BMI in order to avoid impacting physiological variability.

### Initial assessment

The preliminary evaluation was accomplished to examine the eligibility criteria and to obtain descriptive information of the subjects. An anamnesis was initially commenced to confirm the absence of related diseases, the use of medications, apply a questionnaire to consider the level of physical activity, measure blood pressure and to scrutinize the suitability for involvement in the experimental protocol.

The subjects were identified and categorized by gathering age, mass, height, fat percentage, birth weight, delivery mode, HR, respiratory rate (RR), SBP, DBP and BMI. Levels of physical activity for the subjects were arbitrated by applying an IPAQ questionnaire^20^.

The anthropometric measurements were obtained according to Lohman et al.^23^. The mass was determined via a digital scale (W200/5, Welmy, Brazil) with an accuracy of 0.1 kg. Height was measured using a stadiometer (ES2020, Sanny, Brazil) with an accuracy of 0.1 cm. The BMI was computed via the mathematical formula: mass (kg)/height (m^2^). We computed fat percentage through tetrapolar bioimpedance analysis (Omron, Sao Paulo, SP, Brazil).

### Variables, data sources and outcome measures

#### Cardiorespiratory variables

SBP and DBP were verified indirectly by auscultation through a calibrated aneroid sphygmomanometer (Premium, Barueri, SP, Brazil) and stethoscope (Premium, Barueri, SP, Brazil) on the left arm^24^. To avoid misrepresentations, the same researcher measured the cardiorespiratory parameters throughout the entire experiment.

The HR was evaluated by the Polar RS800cx HR monitor (Polar Electro, Finland) and RR measurement was accomplished by counting the respiratory incursions for one minute whilst the subject was unaware of the procedure to reduce the effect of psychological stress on RR^25^.

### HRV analysis

We followed directives from the Task Force of the European Society of Cardiology and the North American Society of Pacing and Electrophysiology^2^ and recommendations from Laborde et al.^26^. The HR was recorded beat-to-beat during the experimental protocol via a HR monitor (Polar RS800cx, Finland) with a sampling rate of 1 kHz. The IBIs recorded were then transferred to the Polar Precision Performance Software (v. 3.0, Polar Electro, Finland) that permits visualization of HR and signal stability. Five-minute intervals were designated for short-term analysis and one-minute interval was selected for ultra-short-term analysis. Intervals were recorded and saved as a ".txt" file. Next, digital filtering was achieved by the same Polar Precision Performance Software supplemented with manual filtering for the elimination of artifacts. For short-term data analysis we selected stable series with 256 IBIs while we enforced 60 IBIs for ultra-short-term analysis. Only series with greater than 95% of sinus beats were included in the study. Further details have been previously documented^27,28^.

The HRV indices applied were the time domain analysis index of the root mean square of successive differences (RMSSD) and the frequency domain index of the high frequency spectral component (HF) of the power spectral density (0.15–0.4 Hz) in absolute units (ms^2^). The aforementioned indices reflect parasympathetic influences on heart rhythm^2^.

We did not enforce low frequency (LF) or the LF/HF ratio since the LF band has been established to be theoretically flawed. The most serious concern is that LF does not index HR sympathetic modulation and so there is a lack of rationale and compelling evidence that its strength in relation to the HF component would index relative strength of vagal and sympathetic signaling^29,30^.

To facilitate calculation of the HRV indices we enforced the Kubios HRV software package (Kubios HRV v.1.1, Biomedical Signal Analysis Group, Department of Applied Physics, University of Kuopio, Finland)^31^.

### Experimental protocols

After the preliminary assessment, the Polar RS800cx (Polar Electro, Finland) strap was placed on the subjects’ chest in the region of the distal third of the sternum. Each experimental protocol was then completed in a single day, all performed on a treadmill.

The subjects underwent treadmill exercise with slope of 1% in the first 5 min for warm up (50–55% of maximal HR (HR_max_): 220 – age)^32^, after that 25 min with increments of 0.5 km/h every minute until attainment of submaximal HR (60–65% of HR_max_). Immediately after exercise, the subjects experienced three minutes standing on the treadmill and then later seated for passive recovery for a further 57 min, totaling 60 min of recovery. During recovery from exercise the subjects remained seated silently with spontaneous breathing. They were unable to complete any movements that would induce autonomic changes, did not sleep or ingest any type of food or drink.

HR, RR, SBP and DBP were measured at the following instants: rest—10th to 15th minute of resting—and during recovery—1st, 3rd, 5th, 7th, 10th, 20th, 30th, 40th, 50th and 60th minutes after exercise.

The ultra-short term RMSSD index was computed at the following moments: rest (M1: 10th to 15th minute of resting) and during recovery: M1 (0 to 1st minute), M2 (1st to 2nd minute) and M3 (2nd to 3rd minute).

**Figure 2:**
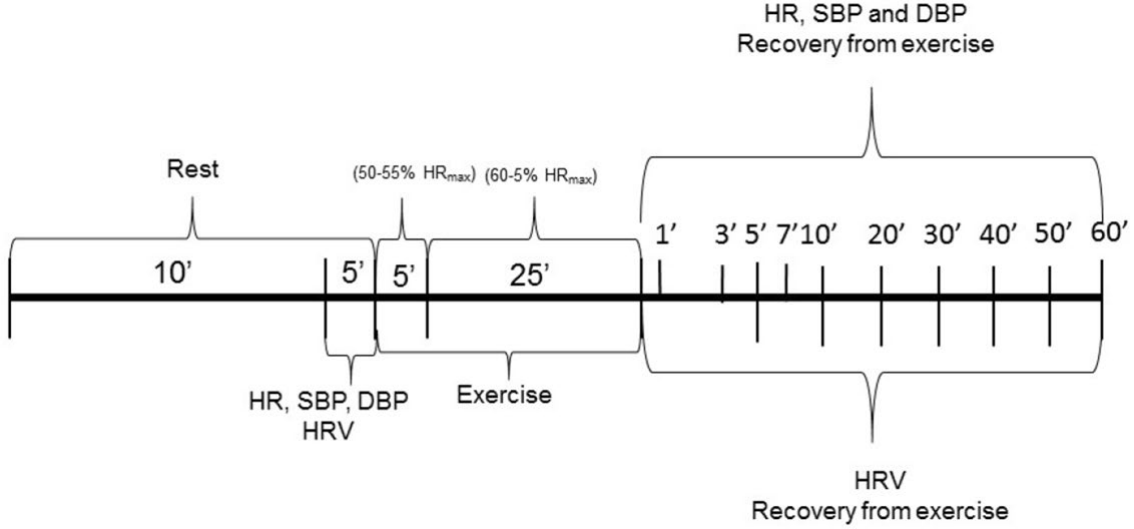
Study design.

The short-term RMSSD and HF indices were taken at the following moments: rest (M1: 10th to 15th minute of resting) and during recovery: M4 (5th to 10th minute), M5 (15th to 20th minute), M6 (25th to 30th minute), M7 (35th to 40th minute), M8 (45th to 50th minute) and M9 (55th to 60th minute)^25,33^ (Fig. 2).

All variables were measured with the subjects in silence, whilst maintaining an empty bladder and undergoing spontaneous breathing.

### Study size

We determined by the sample calculation according to a pilot protocol, in which the online software from the website http://www.lee.dante.br was required based on the RMSSD index. The magnitude of the significant difference assumed was 14.11 ms, considering a standard deviation of 12.8 ms, with alpha risk of 5% and beta risk of 80%. The sample size evaluated, was when the smallest number was 13 individuals per group.

### Statistical analysis

The data normality was evaluated by the Ryan-Joiner test (analogous to the Shapiro–Wilk test). When comparing the descriptive characteristics between the groups, the unpaired Student *t* test (parametric data) or the Mann–Whitney test (non-parametric data) was applied.

We followed strategies for HRV experiment planning, data analysis, and data reporting from Laborde et al.^26^, which suggested that within-subject design has more advantages compared to the between-subject comparison. This is since within-subject design contributes to the elimination of individual differences in respiratory rates and reduces the impact of external variables, including medication, alcohol, smoking, and so forth^34^. Thus, to compare cardiovascular variables and HRV, we applied the repeated measures ANOVA followed by the Bonferroni post-test for parametric distributions or Friedman followed by the Dunn’s post-test for non-parametric distribution.

In an attempt to measure the magnitude of differences, the effect size was calculated via Cohen's *d* and Hedges' *g*, between groups and between two time points. We assumed large effect size for values greater than or equal to 0.9, medium effect size for values between 0.9 and 0.5 and finally, small effect size for values between 0.5 and 0.25^35,36^.

Also, to evaluate the association between birth weight and HRV (RMSSD and HF), the Spearman Correlation test was enforced for non-parametric distributions. Linear Regression analysis was performed to estimate the influences of HRV variables [RMSSD and HF (ms^2^)] between the moments. RMSSD and HF (ms^2^) were considered as dependent variables and the moments were considered as independent variables. The confidence level adopted was 95%.

Statistically significant changes were considered when the “p-value” was less than 0.05 (or, < 5%). We required the Minitab software (Minitab, PA, USA), GraphPad InStat—v3.06, (GraphPad Software, Inc., San Diego California USA) and IBM SPSS Statistics—v22.0 (SPSS Inc. Chicago USA) for the statistical analyses.

## Results

Sample characterization of the 28 subjects analyzed is illustrated in Table 1. The mean age of both groups was comparable. Moreover, there were no statistically significant differences between the groups regarding mass, height, BMI, fat mass and cardiorespiratory variables (HR, SBP, DBP and RR). By design, BW was greater in G2.

**Table 1:** Characterization of the sample regarding age, birth weight, mass, height, body fat, BMI, resting HR, RR, SBP and DBP. Mean ± standard deviation [minimum–maximum].

Variables	G1	G2	p value	Cohen’s d
Birth weight (kg)	2.95 ± 0.19 [2.45–3.16]	3.68 ± 0.31 [3.37–4.5]	< 0.0001	2.83
Mass (kg)	54.35 ± 6.22 [44–65]	58.04 ± 4.51 [51.2–65.3]	0.0765	–
Height (m)	1.63 ± 0.03 [1.56–1.7]	1.64 ± 0.04 [1.56–1.71]	0.6628	–
Body fat (%)	29.59 ± 5.05 [23.5–38.2]	30.12 ± 8.14 [13.5–39.6]	0.7704	–
#Age (years)	20.92 ± 2.99 [18–27]	20.57 ± 2.59 [18–28]	0.8552	–
*BMI (kg/m^2^)	20.29 ± 1.82 [17.9–23.2]	21.55 ± 2.16 [18.9–24.8]	0.0732	–
*SBP rest (mmHg)	103.35 ± 9.74 [82–122]	106.0 ± 9.86 [94–120]	0.444	–
*DBP rest (mmHg)	68.42 ± 7.15 [58–84]	70.85 ± 8.58 [60–84]	0.4549	–
HR rest (bpm)	77.28 ± 11.02 [53–98]	81.64 ± 5.98 [72–98]	0.2156	–
RR (cpm)	14.85 ± 3.65 [11–24]	15.5 ± 3.27 [10–20]	0.4658	–

Average values followed by their respective standard deviations. minimum- and maximum. *BMI* body mass index, *HR* heart rate, *SBP* systolic blood pressure, *DBP* diastolic blood pressure, *RR* respiratory rate, *cpm* cycles per minute, *kg* kilogram, *m* meters, *bpm* beats per minute, *mmHg* millimeters of mercury, *G1* birth weight between 2400 and 3200 g, *G2* birth weight between 3200 and 4500 g.

### Cardiorespiratory variables

**Figure 3:**
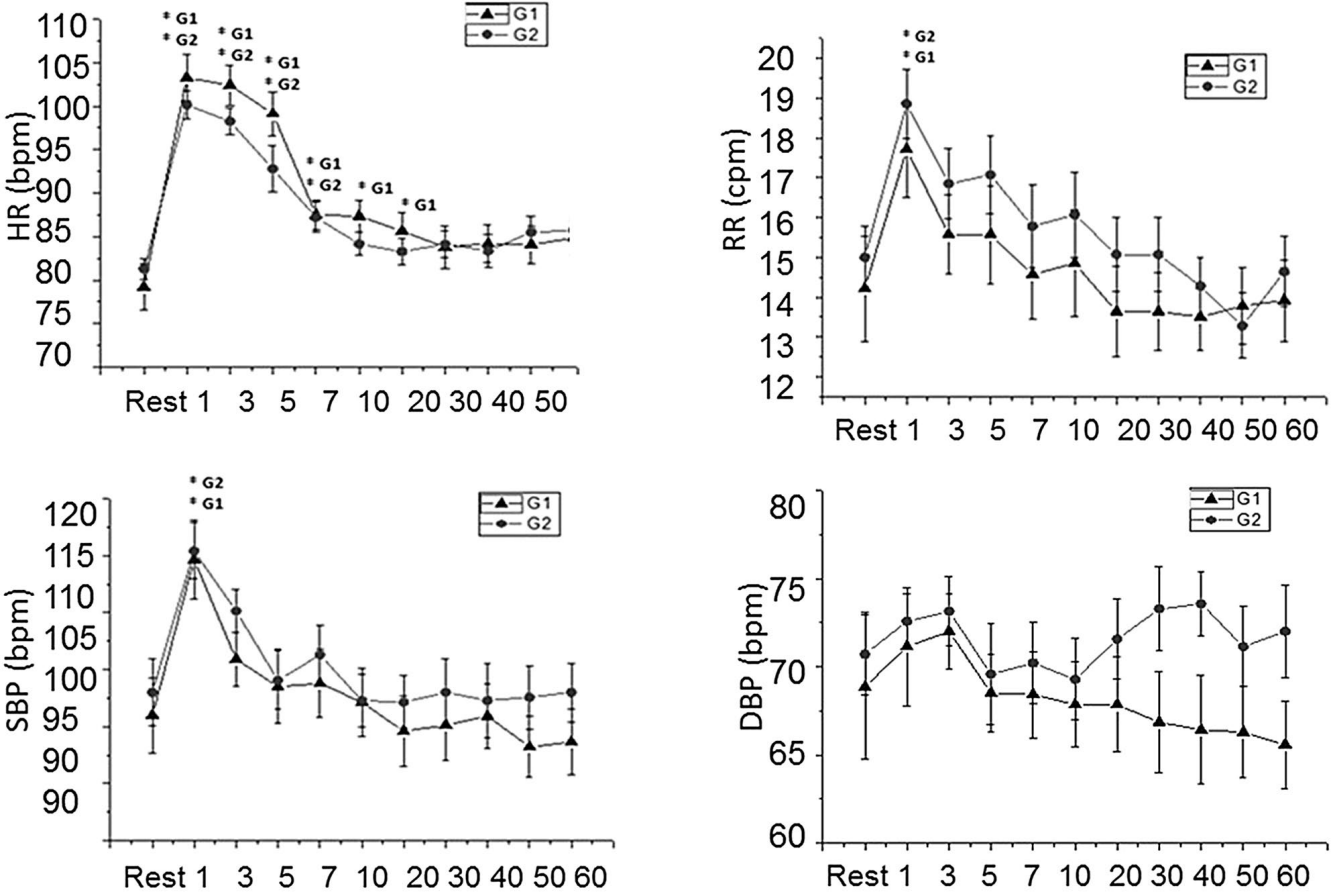
Mean values and respective standard deviations of HR, RR, SBP and DBP obtained at rest and during recovery from moderate aerobic exercise protocol. *G1 and *G2: values with significant differences in relation to rest (p < 0.05) for G1 and G2, respectively. *SBP* systolic blood pressure, *DBP* diastolic blood pressure, *HR* heart rate, *RR* respiratory rate, *mmHg* millimeters of mercury, *bpm* beats per minute, *cpm* cycles per minute, *G1* birth weight between 2400 and 3200 g, *G2* birth weight between 3200 and 4500 g.

HR was greater at the 1st, 3rd, 5th, 7th, 10th and 20th minutes during recovery from exercise compared to rest in G1. In G2, HR was greater at 1st, 3rd, 5th and 7th minutes following exercise compared to rest (Fig. 3).

We observed that RR and SBP were greater at the 1st minute after exercise compared to rest in G1 and G2 (Fig. 3). There was no significant change in DBP between rest and during recovery from exercise in G1 and G2.

### Heart rate variabilities (HRVs)

We detected no significant differences regarding resting RMSSD (p = 0.83), SD1 (p = 0.74) and HF (p = 0.28) between groups.

**Figure 4:**
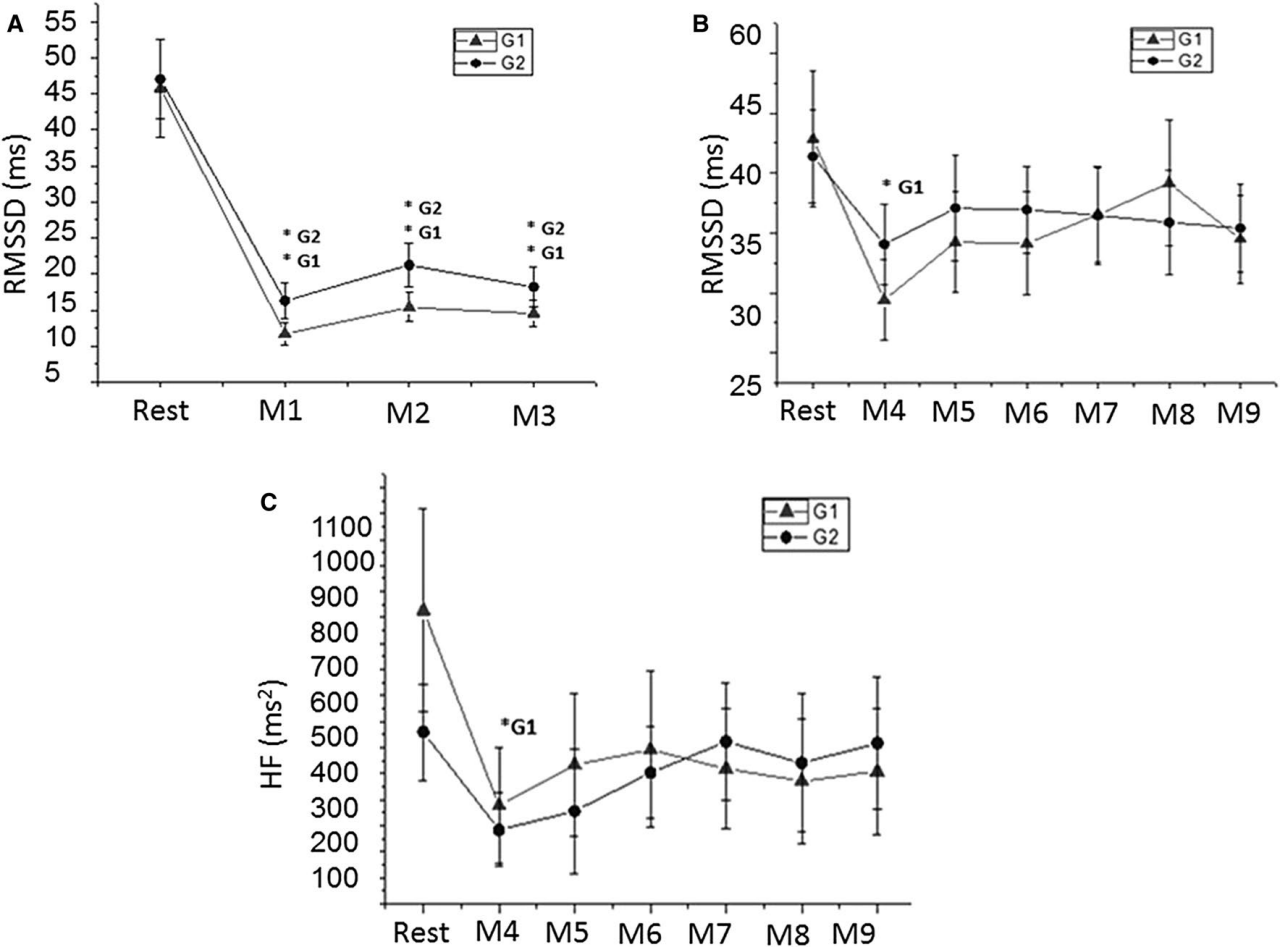
Mean values and respective standard deviations of ultra-short-term RMSSD (**A**), short term RMSSD (**B**) and short-term HF (**C**) obtained at rest and during recovery from moderate aerobic exercise protocol. *G1 and *G2: values with significant differences in relation to rest (p < 0.05) for G1 and G2, respectively. *RMSSD* square root of the square mean of the differences between adjacent normal IBI, *HF* high frequency, *ms* milliseconds, *ms*^*2*^ absolute units, *G1* birth weight between 2400 and 3200 g, *G2* birth weight between 3200 and 4500 g, *Rest* 10th to 15th minute before exercise, *M1* 0 to 1 min, *M2* 1 to 2 min, *M3* 2 to 3 min, *M4* 5th to 10th minute, *M5* 15th to 20th minute, *M6* 25th to 30th minute, *M7* 35th to 40th minute, *M8* 45th to 50th minute and *M9* 55th to 60th during recovery from exercise.

RMSSD, an indicator of ultra-short term HRV, was lower in the initial three minutes following exercise cessation in G1 and G2 (Fig. 4A).

Short-term HRV analysis is revealed in Fig. 4. Regarding short-term HRV, RMSSD (Fig. 4B) and HF (Fig. 4C) were decreased zero to five minutes during recovery from exercise in G1, while no change was recognized in G2.

### Correlation and linear regression

Table 2 illustrates correlation analysis between HRV indices and BW. No significant connection was observed in G1 and G2.

**Table 2:** Correlation between HRV (RMSSD and HF) and birth weight.

Variables	rho	p*
**RMSSD**		
Rest	− 0.072	0.714
Recovery 0–1 min	0.188	0.337
Recovery 1–2 min	0.269	0.165
Recovery 1–3 min	0.132	0.503
Recovery 5–10 min	0.107	0.584
Recovery 15–20 min	0.017	0.929
Recovery 25–30 min	0.173	0.377
Recovery 35–40 min	− 0.053	0.786
Recovery 45–50 min	− 0.157	0.424
Recovery 55–60 min	− 0.014	0.940
**HF (ms**^**2**^**)**		
Rest	− 0.121	0.536
Recovery 5–10 min	− 0.018	0.926
Recovery 15–20 min	− 0.017	0.929
Recovery 25–30 min	0.099	0.615
Recovery 35–40 min	0.078	0.690
Recovery 45–50 min	0.070	0.722
Recovery 55–60 min	0.088	0.654

*RMSSD* root-mean square of differences between adjacent normal RR intervals, *HF* high frequency.

In Table 3, there was a statistically significant association in Model 1 for “1st minute recovery”, “2nd minute recovery”, “3rd minute recovery” and “5–10-min recovery” for G1. In Model 2, we found association for “1st minute recovery” “2nd minute recovery” and “5–10-min recovery”.

**Table 3:** Regression model between RMSSD and HF variables (ms^2^) among moments.

Models	β	95% CI	p	r-adjusted
**Model 1—RMSSD**				
Rest	*Ref*			
Recovery 0–1 min	− 28.12	− 35.38; − 20.86	< 0.001*	0.31
Recovery 1–2 min	− 23.8	− 31.05; − 16.54	< 0.001*	
Recovery 1–3 min	− 25.76	− 33.02; − 18.50	< 0.001*	
Recovery 5–10 min	− 10.37	− 17.63; – 3.11	0.005*	
Recovery 15–20 min	− 6.45	− 13.71; 0.80	0.081	
Recovery 25–30 min	− 6.57	− 13.83; 0.68	0.076	
Recovery 35–40 min	− 5.54	− 12.80; 1.71	0.134	
Recovery 45–50 min	− 4.58	− 11.84; 2.67	0.215	
Recovery 55–60 min	− 7.15	− 14.41; 0.10	0.053	
**Model 2—HF (ms**^**2**^**)**				
Rest	*Ref*			
Recovery 5–10 min	− 281.28	− 523.57; − 38.99	0.023*	
Recovery 15–20 min	− 224.17	− 466.46; 18.10	0.070	
Recovery 25–30 min	− 172.60	− 414.89; 69.68	0.162	
Recovery 35–40 min	− 161.03	− 403.32; 81.25	0.191	
Recovery 45–50 min	− 193.39	− 435.68; 48.89	0.117	
Recovery 55–60 min	− 165.39	− 407.68; 76.89	0.180	

*RMSSD* root-mean square of differences between adjacent normal RR intervals, *HF* high frequency.

## Discussion

We planned to inspect cardiovascular and autonomic recovery after moderate exercise in young healthy term-born women with different BWs. As key results, we observed that: (1) Recovery of HR after exercise cessation was slower in subjects with normal low BW and; (2) HRV return following effort was delayed in subjects with normal low BW. Our data demonstrated that parasympathetic control of heart rhythm during recovery after exercise is improved in women with BW > 3200 g.

Concerning cardiorespiratory variables, we detected SBP and RR increased in the first minute of recovery from exercise in both groups. Regarding DBP, there was no significant difference between rest and post exercise in both groups. Since all subjects in the two groups had similar characteristics, specifically same gender and levels of physical activity, they presented normal physiological behavior during recovery from submaximal aerobic exercise^37^, supporting their healthy state. Based on our study, HR was greater from 0 to 20 min following exercise compared to rest in women with normal low BW. Whereas, HR was higher from zero to seven minutes after exercise cessation in women with normal high BW. We support that recovery of HR was slower in term-born women with normal low BW. Although whilst recent studies analyzed HR recovery in healthy young adults born preterm^17,18^, no previous studies have focused on term-born subjects with normal BW in different BW ranges.

A recent study supports our results, which suggested negative influence of normal low BW on autonomic nervous system. The study conducted by Haraldsdottir et al.^17^ assessed preterm young adults with very normal low BW (< 1500 g), in which recovery was assessed after maximal physical activity with normoxia and hypoxia on an ergometric cycle. Recovery of HR in the initial 2 min of both protocols was slower in preterm infants compared to full term, signifying that very normal low BW impairs autonomic function. This could indicate an increased cardiovascular disease risk. Similarly, Karvonen et al.^18^ hypothesized that preterm birth was related to impaired HR recovery after exercise in young adults. Males and females between 19.9 and 26.3 years old completed a four-minute Åstrand–Ryhming step test. During this exercise test, subjects stepped on and off a bench (40 cm high for men and 33 cm high for women) repeatedly for four minutes at 23 steps per minute influenced by a metronome^38^. The authors specified slower HR recovery in adults born preterm compared to term adults.

Yet, Weitz et al.^39^ evaluated muscle sympathetic nerve activity recordings from the superficial peroneal nerve in subjects with normal low BW (< 2500 g at term) and normal BW (3200–3700 g). These researchers reported lower sympathetic activity in the vasculature of muscles in healthy young people with normal low BW while HR and blood pressure were similar when compared to young people with normal BW. According to the mentioned study, the lower sympathetic activity may have been the outcome of a change in the development of the intrauterine sympathetic autonomic nervous system. Even so, the cardiovascular features may have progressively changed owing to environmental issues such as socioeconomic and/or biological factors.

This explanation is recommended by a longitudinal study performed over six decades, which exhibited that modifiable factors during various stages of life (socioeconomic etc.) can adjust resting HR over the years, even with biological patterns outside the ideal range of development (such as birth weight and size)^40^. An important methodological apprehension regarding the aforesaid studies is the inclusion of either gender and an absence of maintaining levels of physical activity. Both variables have a significant influence on HRV^41,42^.

In this study, we evaluated recovery of HR autonomic regulation after aerobic moderate submaximal exercise. Initial HR values at recovery after physical activity is a significant predictor of mortality. A 6-year longitudinal study by Cole et al.^19^ estimated recovery of HR in individuals without a history of coronary angiography, cardiac surgery, pacemakers, and so forth. Abnormal recovery of HR was considered as a decrease of 12 beats per minute or less from the HR at peak exercise. The researchers found that 9% of the sample deceased from various causes, but within this 9%, 56% described the slower recovery of HR in the first minute after exercise.

Thus, we detected a significant reduction of HF (ms^2^) and short-term RMSSD from 5 to 10 min after exercise cessation compared to rest in women with normal low BW, but this was not observed in women with normal high BW group. Taken together, our data suggests that term-born women with normal low BW achieved slower HRV recovery compared to those with normal high BW. This is because RMSSD and HF indices required longer to attain values approximating to rest. Both indices correspond to parasympathetic modulation of heart rhythm, indicating delayed vagal return in women with normal low BW. It is worth emphasizing that consistent with Terziotti et al.^43^ there is perseverance in the vagus nerve flow restriction until almost 15 min following exercise termination. We highlight that even though women with normal low BW had delayed recovery compared to women with normal high BW, both groups presented normal physiological responses.

Specific cellular mechanisms may be theorized to explain the delayed HRV recovery from exercise in term-born women with normal low BW. The immune system and antioxidant status are immature in preterm infants. Hypoxic stress during birth leads to greater production of free radicals and DNA damage in preterm infants and is related to gestational age and BW^44^. Similarly, Mirzarahimi et al.^45^ established that umbilical cord blood antioxidant levels are higher in healthy term newborns with normal high BW. The aforesaid factors are suggested to be involved in increased cardiovascular risk in preterm adults. In this way, we may suggest that blood cord immunological and antioxidant status are better in women with BW > 3200 g compared to women with BW < 3200 g.

Some other points are worth highlighting. Although we found no significant difference between groups regarding resting HF values, we detected that it tended to be higher in subjects with lower BW. Yet, this finding indicates that parasympathetic withdrawal was more intense in this group, supporting that subjects with lower BW presents delayed autonomic recovery. Additionally, high HF values in the lower BW groups underpin that this group was comprised of healthy individuals. We did not estimate the greatest time for breast-feeding; yet, all were breast-fed at least for 6 months after birth. We studied young adult term-born physically active women that eliminates potential sources of bias, reduces the unpredictability of the variables, enhances reproducibility, improves physiological and clinical interpretation. In this manner, our results cannot be transferred to subjects with other medical conditions. While we investigated a small sample (14 per group) owing to a strong methodological criterion, statistical analysis provided significance. But we encourage further studies to investigate whether BW > 3200 g is more beneficial than BW < 3200 g.

We reveal important results to the clinical research community, since cardiovascular disorders are projected to be one of the key public health challenges globally in the twenty-first century^46^. Moreover, impaired autonomic function is consistent with significantly higher rates of cardiovascular disease and mortality^47,48^. Our results advocate that young healthy young women born term with BW below 3200 g may be at a slightly increased risk of developing cardiovascular disease compared to those with BW > 3200 g. Thus, we reinforce the importance of preventive measures for children with BW < 3200 g during their whole lifetime. These results highlight and promote preventive strategies, cooperating with clinicians to decrease cardiovascular morbidity and mortality.

## Conclusion

Young adult healthy term-born women with normal low BW exhibited slower recovery from moderate exercise compared to subjects with normal high BW. We emphasize this result for medical clinicians employed to achieve maximum beneficial and preventive strategies.
